# Absolute and lung volume-normalized deep learning-derived pulmonary vascular measures in idiopathic pulmonary fibrosis: distinct clinical associations and the role of lung volume in prognosis

**DOI:** 10.3389/fmed.2026.1833470

**Published:** 2026-09-29

**Authors:** Gaetano Rea, Stefano Sanduzzi Zamparelli, Annalisa Mazzocca, Roberta Lieto, Dario Bruzzese, Bruno Crestani, Marialuisa Bocchino

**Affiliations:** 1 Radiology Unit, Azienda Ospedaliera dei Colli, Monaldi Hospital, Naples, Italy; 2 Division of Pneumology and Semi-Intensive Respiratory Therapy, AORN A. Cardarelli, Naples, Italy; 3 Department of Clinical Medicine and Surgery, Federico II University, Naples, Italy; 4 Respiratory Medicine Unit, Azienda Ospedaliera dei Colli, Naples, Italy; 5 Medical Statistics Unit, Department of Public Health, Federico II University, Naples, Italy; 6 Department of Pneumology, Bichat-Claude Bernard AP-HP, Université Cité Paris, Paris, France; 7 Department of Advanced Biomedical Sciences, Federico II University, Naples, Italy

**Keywords:** idiopathic pulmonary fibrosis, prognosis, pulmmonary vascular, quantitative CT, survival

## Abstract

**Introduction:**

Idiopathic pulmonary fibrosis (IPF) has an unpredictable course and poor outcome, and lung function-based risk stratification remains incomplete. Whether absolute and lung volume-normalized deep learning-derived CT pulmonary vascular measurements show distinct associations with disease severity and mortality in patients with IPF remains unclear.

**Methods:**

In this dual-center retrospective study, 118 adults with newly diagnosed IPF, including 57 from the Italian cohort and 61 from the French cohort, underwent baseline chest CT analyzed with a deep learning-based quantitative pipeline combining lung parenchyma (whole lung volume, WLV; fibrosis extent) and pulmonary vascular metrics (total blood volume, TBV; and derived BV_5_ and BV_>10_ indices representing vessels with cross-sectional areas of <5 mm² and >10 mm²). Twenty subjects without lung disease served as a comparison group. Associations with fibrosis were assessed by Pearson correlation, mortality by multivariable Cox hazards models, and longitudinal lung function by linear mixed-effects models. Additional models examined the prognostic associations of vascular metrics after accounting for WLV.

**Results:**

Compared with controls, IPF patients had lower WLV and higher absolute TBV. Relative TBV (normalized to WLV) correlated strongly with fibrosis, while absolute TBV showed weaker associations. Relative vascular metrics inversely related to baseline lung function, whereas absolute TBV showed no significant association. Baseline TBV, BV_5_, and BV_>10_ did not predict forced vital capacity (FVC) or diffusing capacity of the lung for CO trajectories, but higher WLV predicted better FVC over time. Over a median 7.8-year follow-up, 72 deaths occurred. In multivariable models, higher absolute TBV, lower-lobe BV, BV_5_, BV_>10_, and WLV, but not normalized TBV, were associated with reduced mortality after adjustment for established clinical variables. These findings suggest that preservation of lung volume and pulmonary vascular volume may be associated with improved survival. However, the associations of absolute vascular metrics with mortality were substantially attenuated after additional adjustment for WLV.

**Conclusion:**

Normalized and absolute pulmonary vascular metrics capture different aspects of IPF, with normalized measures associated with fibrosis burden and functional impairment, while absolute vascular volumes were associated with mortality, although this association was substantially attenuated after accounting for WLV. Absolute vascular volume may therefore partly reflect preserved lung volume rather than an independent prognostic effect. Prospective validation is warranted.

## Introduction

Idiopathic pulmonary fibrosis (IPF) is the most studied form of interstitial lung disease (ILD) and is characterized by distinct pathogenetic mechanisms and aggressive clinical behavior. Despite more than a decade of antifibrotic therapies, prognosis remains poor and the disease course highly variable (1). Routine monitoring relies on pulmonary function tests, particularly forced vital capacity (FVC) and diffusing capacity of the lung for carbon monoxide (DLCO). The combination of these measures with demographic variables led to the development of GAP (Gender-Age-Physiology) score, a widely used prognostic model (2). However, this index does not fully capture the marked heterogeneity of IPF, highlighting the need for additional biomarkers.

Imaging has become central to the diagnosis and prognosis of fibrotic ILDs. Quantitative CT approaches allow objective evaluation of structural lung abnormalities and have shown that automated software can reliably quantify fibrotic burden and identify imaging features associated with clinical outcomes (3, 4). More recently, attention has shifted toward the pulmonary vascular compartment as a potential contributor to fibrosis development and disease progression. The pulmonary circulation undergoes structural and functional remodeling in chronic lung diseases and may ultimately lead to pulmonary hypertension, with aging being a contributing factor (5). Several CT-based studies have reported that pulmonary total blood volume (TBV), often expressed relative to whole lung volume (WLV), is increased in IPF (6–11). Proposed mechanisms include redistribution of blood flow toward relatively preserved lung regions, mechanical traction on vessels caused by fibrotic remodeling, and the formation of pleuroparenchymal or bronchopulmonary arterial anastomoses (12–14). Quantitative analyses have further suggested that a TBV increase exceeding 5% is independently associated with higher mortality risk in IPF (6). In the pooled PROFILE cohort, deep learning-based CT segmentation demonstrated that lung, vascular, and fibrosis volumes were associated with overall and progression-free survival (15). Some authors have even hypothesized that vascular alterations may precede fibrosis, supporting a possible pathogenic role of the pulmonary vasculature in IPF (16–18).

In this context, we aimed to directly compare absolute and normalized pulmonary vascular volumes derived from a deep learning-based quantitative CT algorithm to determine whether they capture different aspects of IPF severity and show distinct associations with fibrosis burden, functional impairment, longitudinal lung function decline, and mortality. Multivariable analyses were performed to evaluate mortality associations after adjustment for established clinical and radiological variables.

## Materials and methods

### Study population

The study included adults (>18 years) with newly diagnosed IPF, recruited between January 2015 and December 2022 from two tertiary referral centers: Federico II University (Naples, Italy) and Bichat Hospital (Paris, France). Diagnosis followed contemporary consensus guidelines (1, 19, 20). Non-IPF ILDs were excluded. Additional exclusion criteria were: recent acute exacerbation, active malignancy, ongoing anti-fibrotic therapy, follow-up <1 year, incomplete data, inadequate imaging (including slice thickness >1.5 mm, significant motion artifacts, poor breath-hold, non-HRCT acquisition protocols, use of intravenous contrast media, or reconstruction with sharp/bone kernels only), or non-usual interstitial pneumonia (UIP) CT patterns. Baseline and follow-up pulmonary function tests included forced vital capacity (FVC) and hemoglobin-adjusted single-breath DLCO (21, 22). The GAP scores were calculated according to Ley et al. (2). Demographics, clinical, functional, and imaging data were anonymized and stored in a secure database. The control group consisted of 20 subjects retrospectively selected from our institutional database. These individuals had undergone chest CT for indications unrelated to pulmonary disease and showed no evidence of lung pathology. Controls were selected, whenever possible, to match the study population for age and sex. The study was approved by the local ethics committee (protocol AOC/0006273/2025), and informed consent was obtained; for deceased patients without representatives, consent was waived. Cause of death was determined from hospital records or family reports.

### Chest CT imaging and preprocessing

Baseline volumetric high-resolution CT scans were retrospectively collected using five CT systems (Siemens Somatom Force and Drive, GE LightSpeed VCT 64-slice, Toshiba Aquilion One, and Philips Brilliance 64-slice). Acquisition parameters included 120 kVp with automatic tube current modulation, rotation times of 0.25–0.5 s, and slice thicknesses of 0.6–1.25 mm. Images were reconstructed with soft-tissue and high-resolution lung kernels using matrices of 512 × 512 to 1,024 × 1,024. CTDIvol ranged from 1.2 to 5.5 mGy following ALARA principles. Scans were obtained in full inspiration with the patient supine. Scans meeting the predefined exclusion criteria described above were excluded. Although imaging data were acquired using five different CT scanners, all examinations followed standardized high-resolution CT acquisition protocols with consistent reconstruction parameters (including soft kernels and similar slice thickness ranges), ensuring methodological comparability across datasets. Technical details are reported in Supplementary Table S1. No *post-hoc* statistical harmonization or formal scanner-effect correction was applied.

### Visual assessment

Two thoracic radiologists (G.R, R.L.) confirmed UIP or probable UIP patterns according to current guidelines (1). This step was performed prior to quantitative analysis. Radiologists were blinded to clinical outcomes, longitudinal pulmonary function data, and survival information during image review.

### Deep learning-based quantitative analysis

#### Lung parenchyma analysis

All images were reconstructed using soft kernels for deep learning-based lung parenchyma analysis. Measurements were performed using the AVIEW deep learning platform (Coreline Soft, Seoul, Korea). Deep learning-based lung parenchyma analysis was performed after automated lung parenchyma segmentation, excluding thoracic wall and central airways, with five-lobe subdivision via deep learning-based fissure detection. Each voxel was classified using a deep-learning model into normal lung, ground-glass opacity, consolidation, emphysema, reticulation, or honeycombing; fibrosis extent was defined as the sum of reticulation and honeycombing. Automated outputs were visually reviewed and corrected by two thoracic radiologists (G.R., R.L.). This review was performed as a simultaneous consensus quality-control process, rather than as two independent manual segmentations followed by *post-hoc* adjudication. Minor manual corrections were required in 5 of 118 cases (4.2%) and were limited to small peripheral subpleural areas in which paraseptal emphysema and honeycombing cysts showed overlapping attenuation patterns, occasionally affecting automated voxel classification. In these few cases, ambiguous regions were discussed and refined in real time by consensus. No formal reader discrepancies or separate segmentation tracks were therefore generated. Because independent segmentations were not performed, formal inter-reader variability metrics were not available. No further manual modifications were made to the final quantitative measurements.

#### Pulmonary vascular analysis

TBV analysis used automated vessel segmentation implemented within the AVIEW platform (Coreline Soft, Seoul, Korea), excluding non-vascular structures. TBV was calculated both as an absolute value and normalized to whole lung volume (WLV; TBV/WLV). Volumetric and cross-sectional metrics included absolute values of BV₅ and BV_>_₁₀ (<5 mm² and >10 mm², respectively), as well as their ratios to TBV (BV₅/TBV and BV_>_₁₀/TBV). While TBV was available at both whole-lung and lobar levels, BV₅ and BV_>_₁₀ were generated only at the whole-lung level and were therefore not evaluated regionally. The AVIEW vascular pipeline combines deep learning-based segmentation with conventional image-processing algorithms. A 3D U-Net architecture is first used to generate a vascular mask, which is subsequently refined using multiscale Hessian filtering and graph-based 3D reconstruction to extract vessel centerlines and ensure continuity of the vascular tree. This hybrid framework enables automated and robust vascular quantification, particularly in fibrotic lungs with substantial parenchymal distortion.

### Statistical analysis

Standard descriptive statistics summarized the cohorts: mean ± standard deviation or median (interquartiles, IQR) for numerical variables, and counts with percentages for categorical variables, without reporting *p*-values and using standardized mean differences (SMDs) to quantify the magnitude of between-cohort differences (23). Pearson correlation assessed relationships between TBV-derived measures and fibrosis extent; in addition, partial Pearson correlations adjusted for age at diagnosis and sex were computed to assess whether the associations between vascular metrics and fibrosis extent were influenced by demographic or anthropometric factors. Univariate and multivariable Cox models evaluated the prognostic impact of pulmonary vascular measures on mortality, reported as hazard ratios (HRs) with 95% confidence intervals (CIs). Longitudinal FVC% and DLCO% trajectories were modeled with linear mixed-effects models including time, baseline TBV measures, and their interaction; non-significant interactions were excluded. Analyses were performed using R (version 4.4.0, https://www.r-project.org/).

## Results

### Study population

A total of 118 of 279 patients with newly diagnosed IPF were included in this retrospective cohort study: 57 from the Italian center and 61 from the French center. According to the study protocol, 161 patients were excluded for not meeting eligibility criteria (Figure 1). Twenty control individuals (mean age 63.5 ± 4.9 years; 13 males, 65%) served for descriptive comparisons of WLV and TBV. The main demographic and clinical characteristics of the study cohorts are summarized in Table 1. Mean age at diagnosis ranged from 68.7 to 71.2 years, and males predominated in both cohorts. Smoking status, body mass index, and prevalence of dyslipidemia were comparable between cohorts, whereas a family history of ILD and diabetes were more frequent in the French cohort. Radiologic patterns (UIP *vs.* probable UIP) and baseline pulmonary function parameters were also similar. GAP stage distribution was broadly comparable between cohorts, with severe cases accounting for <10% overall. Antifibrotic treatment differed modestly between centers: pirfenidone was more frequently used in the Italian cohort, whereas pirfenidone and nintedanib were evenly distributed in the French cohort. Fewer than 15% of patients remained untreated. Overall, most demographic and clinical characteristics showed small-to-moderate between-cohort differences, whereas larger differences were observed for antifibrotic treatment patterns.

**Figure 1:**
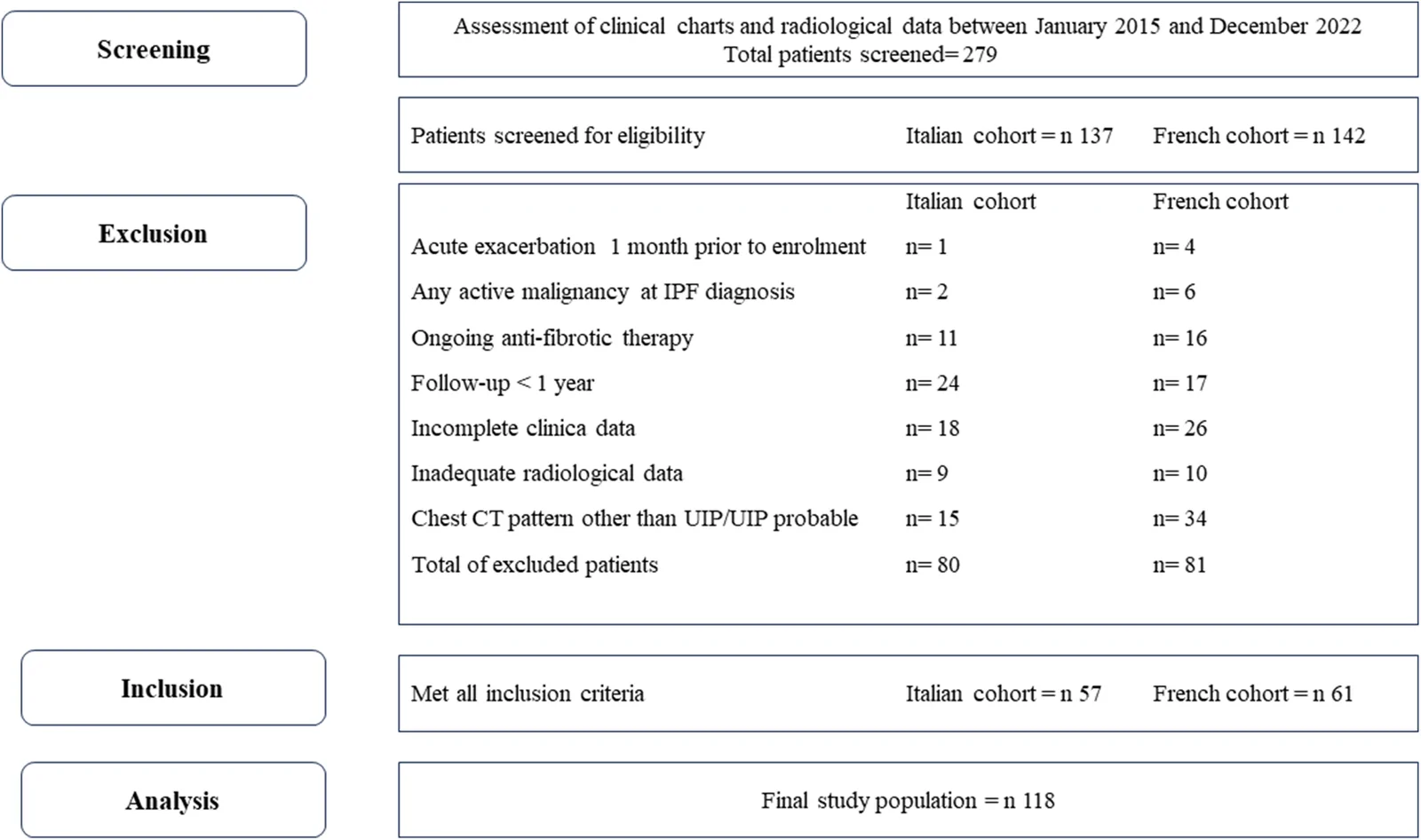
Flow diagram illustrating the selection of patients eligible for the study. The diagram details the number of patients assessed for eligibility, excluded, and ultimately included in the analysis, along with reasons for exclusion at each step.

**Table 1:** Demographics and clinical features of the study population.

Characteristics	Overall population (*n* = 118; 100%)	Italian cohort (*n* = 57; 48.3%)	French cohort (*n* = 61; 51.7%)	SMD
Demographics				
Sex				
Males	91 (77.1)	41 (71.9)	50 (82)	0.240
Age at diagnosis, years	70 ± 8.4 (38 to 87)	68.7 ± 8.5 (38 to 83)	71.2 ± 8.2 (51 to 87)	0.297
Risk factors				
Smoking habit	87 (74.4)	44 (77.2)	43 (71.7)	0.127
Pack/year	25 [20; 35] (5 to 70)	25 [30; 45] (10 to 70)	25 [20; 40] (5 to 60)	0.174
BMI, kg/m^2^	27 [24.2; 30] (16.4 to 45)	27.5 [24.5; 30] (16.4 to 39.8)	26.1 [24.2; 30.1] (16.7 to 45)	0.127
ILD familiarity	13 (11)	3 (5.3)	10 (16.4)	0.364
Dyslipidemia	46 (38.7)	22 (37.9)	24 (39.3)	0.015
Main comorbidities				
Ischemic heart disease	26 (21.8)	11 (19)	15 (24.6)	0.128
Systemic arterial hypertension	65 (54.6)	33 (56.9)	32 (52.5)	0.074
Type II diabetes	39 (32.8)	15 (25.9)	24 (39.3)	0.280
Chest CT pattern				0.162
UIP	51 (43.2)	27 (47.4)	24 (39.3)	
Probable UIP	67 (56.8)	30 (52.6)	37 (60.7)	
Lung function				
FVC (% pred)	84.3 ± 26.4 (36 to 171)	82.6 ± 30.7 (36 to 171)	85.9 ± 21.9 (46 to 143)	0.123
FEV_1_ (% pred)	85.3 ± 30.6 (36 to 171)	83.0 ± 31.3 (46 to 171)	87 ± 30.4 (36 to 155)	0.130
Hb-adjusted DLCO_sb_ (% pred)	52.2 ± 16.2 (16 to 92)	51 ± 16.7 (16 to 92)	53.3 ± 15.7 (23 to 90)	0.144
Disease severity				0.320
GAP I	61 (51.7)	25 (43.9)	36 (59)	
GAP II	45 (38.1)	26 (45.6)	19 (31.1)	
GAP III	12 (10.2)	6 (10.5)	6 (9.8)	
Anti-fibrotic therapy				0.745
None	16 (13.6)	8 (14)	8 (13.1)	
Nintedanib	33 (28)	17 (29.8)	16 (26.2)	
Pirfenidone	47 (39.8)	29 (50.9)	18 (29.5)	
Both	22 (18.6)	3 (5.3)	19 (31.1)	

BMI, body mass index; ILD, interstitial lung disease; UIP, usual interstitial pneumonia; FVC, forced vital capacity; FEV_1_, forced expiratory volume in 1 s; Hb, hemoglobin; DLCO_sb_, diffusing capacity of the lung for carbon monoxide_single−breath_; GAP, Gender-Age-Physiology; SMD, standardized mean difference.

Categorical variables are reported as number (percentage). Continuous variables are expressed as mean ± standard deviation (SD) and range (min-max), or as median [interquartile range] and range (min-max), where appropriate. Between cohort differences are reported as standardized mean differences (SMDs).

### Relative vascular metrics correlated with fibrosis extent

Table 2 reports deep learning-based estimates of lung parenchyma metrics (WLV and fibrosis extent) in each cohort and in the overall study population. WLV was lower in the Italian compared with the French cohort, although values in the entire IPF population remained markedly lower than in the comparison group [4,048 ± 1,113 cm^3^ (1,767–6,418) *vs.* 5,431 ± 1,124 cm^3^ (3,514–8,183)]. Whole-lung fibrosis extent was slightly higher in Italian individuals. As expected, fibrotic abnormalities showed a clear basal predominance, with the highest fibrosis extent observed in the lower lobes (Supplementary Table 2A). Absolute TBV values were higher in IPF than in controls [322 ± 95 cm^3^ (60–566) vs. 235.7 ± 47.9 cm^3^ (131.4–318.7)]. Similarly, relative TBV values were also higher in IPF than in controls [8.3 ± 2.6% (2.2–16.6) *vs.* 4.4 ± 0.8% (3.1–5.4)]. Details of pulmonary vascular metrics are reported in Table 2. TBV was available as both whole-lung and lobar measurements (Supplementary Table 2B), whereas BV_5_ and BV_>10_ metrics were generated only at the whole-lung level. Several pulmonary vascular metrics differed between the Italian and French cohorts, with standardized mean differences indicating moderate-to-large between-cohort differences. Figure 2 illustrates a representative example of the deep learning-based quantification of fibrotic alterations and pulmonary vascular volume. For subsequent analyses, patients from the two cohorts were pooled, while accounting for cohort membership in multivariable analyses. Correlation analysis showed that WLV was inversely associated with fibrosis score (*r* = −0.45; 95% CI: −0.58 to −0.29) (Table 3). Relative TBV demonstrated a strong positive correlation with fibrosis extent (*r* = 0.65, 95% CI: 0.54 to 0.75), whereas the association was weaker when TBV was expressed as an absolute value (*r* = 0.23, 95% CI: 0.03 to 0.37). BV_5_ and BV_>10_ metrics were also positively correlated with fibrosis extent, both as relative and absolute measures (Table 3). To account for the potential confounding effects of age at diagnosis and sex, partial correlations were also computed. The adjusted coefficients were highly consistent with the corresponding crude Pearson correlations, with only minor attenuation observed for absolute vascular measures (Table 3). When evaluating the associations between vascular metrics and fibrosis extent at the lobar level, although the magnitude of the correlations varied across lobes, relative vascular metrics consistently showed stronger associations with fibrosis extent than the corresponding absolute measures (Supplementary Table S3).

**Table 2:** Quantitative assessment of fibrosis extent and vascular metrics in the study population.

Parameter	Overall population *n* = 118 (100%)	Italian cohort *n* = 57 (48.3%)	French cohort *n* = 61 (51.7%)	SMD
WLV, cm^3^	4,048 ± 1,113 (1,767 to 6,418)	3,755 ± 1,132 (1,767 to 6,418)	4,323 ± 1,031 (2,417 to 6,367)	0.523
Fibrosis score, %	13 [6.2; 23.8] (1 to 49)	16 [8.5; 31.5] (2 to 49)	10 [4.5; 18] (1 to 38)	0.610
TBV, cm^3^	322 ± 95 (60 to 566)	303.8 ± 97 (60 to 547)	339 ± 90.7 (98 to 566)	0.375
TBV, %	8.3 ± 2.6 (2.2 to 16.6)	8.5 ± 3 (2.2 to 16.6)	8.1 ± 2.2 (3.7 to 14.9)	0.166
BV_5_, cm^3^	161.25 ± 63.55 (17 to 319)	143.54 ± 62.98 (17 to 306)	177.8 ± 59.98 (38 to 319)	0.622
BV_5_, %	48.55 ± 8.34 (28.33 to 64.72)	45.16 ± 8.31 (28.33 to 62.58)	51.71 ± 7.09 (38.78 to 64.72)	0.905
BV_>10_, cm^3^	261.89 ± 84.16 (43 to 485)	242.68 ± 85.1 (43 to 460)	279.84 ± 79.84 (79 to 485)	0.499
BV_>10_, %	80.35 ± 5.05 (66.02 to 89.66)	78.28 ± 4.93 (66.02 to 87.5)	82.28 ± 4.39 (71.63 to 89.66)	0.901

WLV, whole lung volume; TBV, total blood volume; BV, blood volume; SMD, standardized mean difference.

Data are presented as mean ± standard deviation (SD) and range (min-max), or as median [interquartile range] and range (min-max), where appropriate. Between cohort differences are reported as standardized mean differences (SMDs).

**Figure 2:**
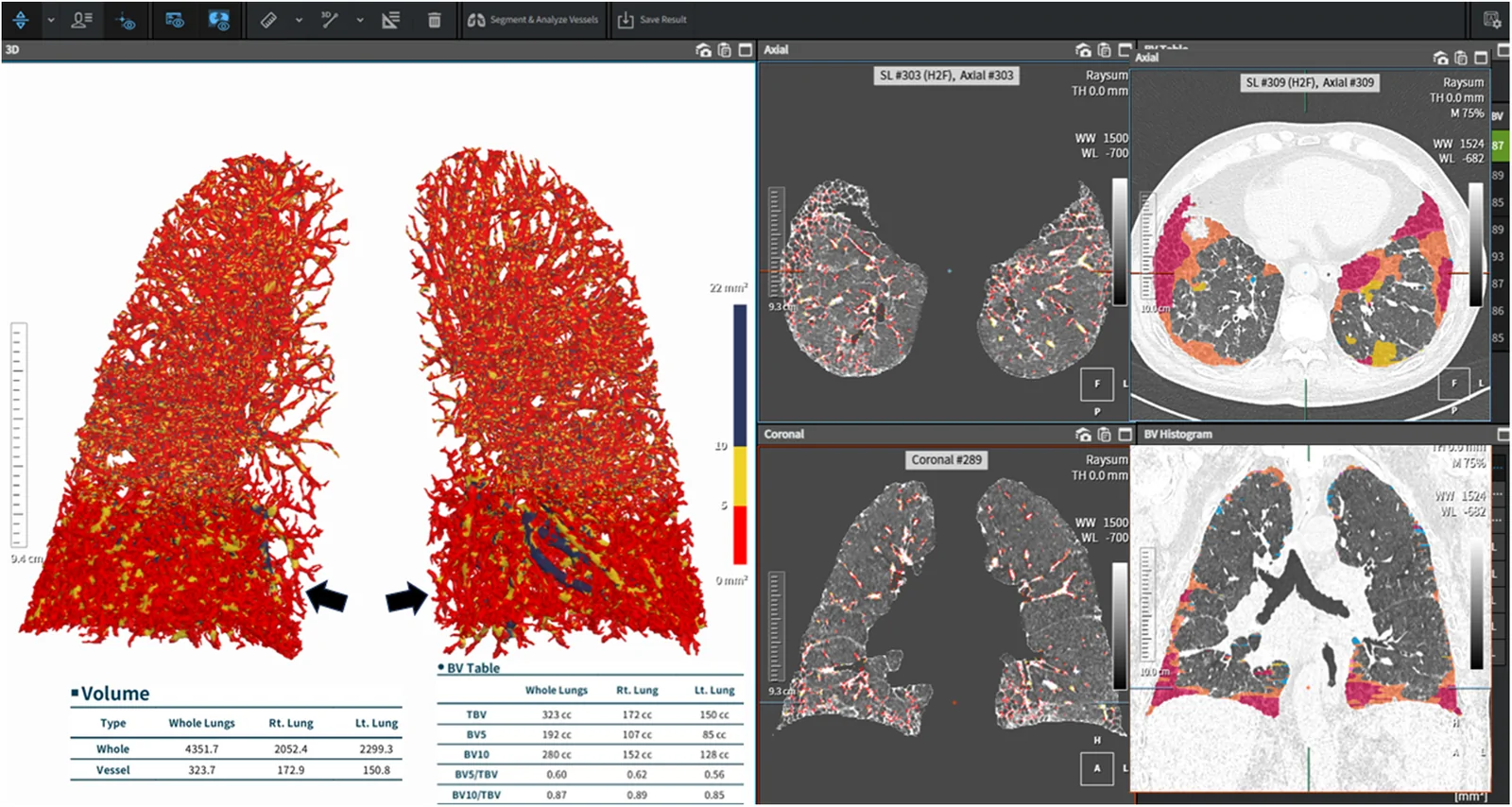
Representative three-panel visualization of pulmonary vasculature and parenchymal fibrotic deep-learning (DL) combined analysis in an 80-year-old male patient with idiopathic pulmonary fibrosis. Left: DL-based segmentation and three-dimensional reconstruction of the pulmonary vascular tree, demonstrating diffuse and pronounced vascular arborization with greater representation in the basal regions (black arrows). Center: Multi-planar volumetric renderings of the pulmonary vasculature in axial (upper row, lower lobes) and coronal (bottom row) orientations, illustrating vessel distribution, density, and spatial relationships to fibrotic parenchyma. Right: Schematic overlay of parenchymal patterns and vascular segmentation from a representative chest CT slice. The axial view (top) highlights marked lower-lobe involvement, whereas the coronal view (bottom) aligns these parenchymal abnormalities with the corresponding vascular reconstructions. Estimates of quantitative analysis: Whole lung volume (WLV), 4.351.7 cm^3^; fibrosis extent, 16%; total blood volume (TBV), 323 cm^3^; TBV/WLV, 7.4%.

**Table 3:** Correlations and partial correlations between pulmonary blood volume-derived measures and fibrosis extent.

	Fibrosis extent (%)			
Parameter	Crude Pearson *r* (95% CI)	*p*	Partial Pearson *r* (95% CI)	*p*
WLV, cm^3^	−0.45 (−0.58 to −0.29)	**<0**.**001**	−0.56 (−0.67 to −0.42)	** <0**.**001**
TBV, %	0.65 (0.54 to 0.75)	**<0**.**001**	0.65 (0.52 to 0.74)	** <0**.**001**
TBV, cm^3^	0.21 (0.03 to 0.37)	**0**.**023**	0.14 (−0.04 to 0.32)	0.13
BV_5_, %	0.26 (0.08 to 0.42)	**0**.**005**	0.22 (0.03 to 0.38)	**0**.**02**
BV_5_, cm^3^	0.28 (0.11 to 0.44)	**0**.**002**	0.22 (0.04 to 0.39)	**0**.**016**
BV_>10_, %	0.25 (0.07 to 0.41)	**0**.**007**	0.23 (0.04 to 0.39)	**0**.**015**
BV_>10_, cm^3^	0.26 (0.08 to 0.42)	**0**.**005**	0.20 (0.01 to 0.37)	**0**.**035**

WLV, whole lung volume; TBV, total blood volume; BV, blood volume.

Data are presented as Pearson's and partial Pearson's correlation coefficients with 95% confidence intervals. Partial correlations were adjusted for age at diagnosis and sex. Associations with a *p* value <0.05 were considered statistically significant and reported in bold.

As expected, pairwise correlations among WLV and pulmonary vascular metrics were generally moderate to very strong (Supplementary Table S4), reflecting the substantial biological and mathematical overlap between these measures. The only exceptions were the relative BV_5_ and BV_>10_ metrics, which were not significantly correlated with WLV.

### Relative vascular metrics were inversely related to baseline lung function but had no impact during follow-up

At baseline, WLV was positively correlated with lung function parameters (*r* = 0.323 for FVC% predicted and *r* = 0.471 for DLCO%, both *p* < 0.001). In contrast, normalized TBV was inversely correlated with FVC (*r* = −0.444; *p* < 0.001) and DLCO (*r* = −0.511; *p* < 0.001), whereas no significant correlations were observed when TBV was expressed as an absolute value. Relative BV_5_ and BV_>10_ showed modest inverse correlations with both FVC and DLCO, while similar trends were observed for absolute measures but did not reach statistical significance (Supplementary Table S5). In a subgroup of 94 patients (median follow-up 2.8 years), linear mixed-effects models were used to assess longitudinal decline in FVC and DLCO. After adjustment for baseline lung function, cohort, sex, age at diagnosis, GAP stage, UIP pattern, and fibrosis extent, neither TBV nor its interaction with time was significantly associated with functional decline. These findings were consistent regardless of whether vascular metrics were expressed as relative or absolute values. By contrast, higher baseline WLV was associated with higher FVC values during follow-up (coefficient = 0.58% per 100 mL; *p* < 0.001), whereas no association was observed for DLCO.

### Absolute vascular metrics were associated with lower mortality, with associations attenuated after accounting for whole lung volume

During a median follow-up of 7.8 years (95% CI: 7.6 to 8.7), 72 deaths were recorded, with a median overall survival of 5.3 years (95% CI: 4.3 to 8.6). The main causes of death were disease progression (36.4%), acute exacerbation (10.2%), cardiovascular or cerebrovascular events (6.8%), pulmonary embolism (1.7%), and lung cancer (5.9%).

In univariate analyses, GAP stage, UIP pattern, fibrosis extent, and relative TBV were significant predictors of mortality, whereas WLV showed a protective effect (HR 0.95 per 100 cm^3^ increase, 95% CI: 0.93 to 0.98; *p* < 0.001) (Supplementary Table S6). In multivariable models including cohort, gender, age at diagnosis, fibrosis extent, chest CT pattern, BMI and GAP score, neither fibrosis extent nor TBV% retained an independent prognostic association. In contrast, absolute TBV, lower-lobe BV, and the derived vascular metrics BV_5_ and BV_>10_, together with WLV, remained independently associated with reduced mortality, indicating a protective effect (Figure 3A and Supplementary Table S7A). When WLV was additionally included in the models, the associations of absolute vascular metrics with mortality were substantially attenuated toward the null, whereas effect estimates for relative vascular metrics remained largely unchanged (Figure 3B). Notably, WLV remained associated with lower mortality in models including relative vascular metrics, while its association was weaker when modeled jointly with absolute vascular volumes (Supplementary Table S7B).

**Figure 3:**
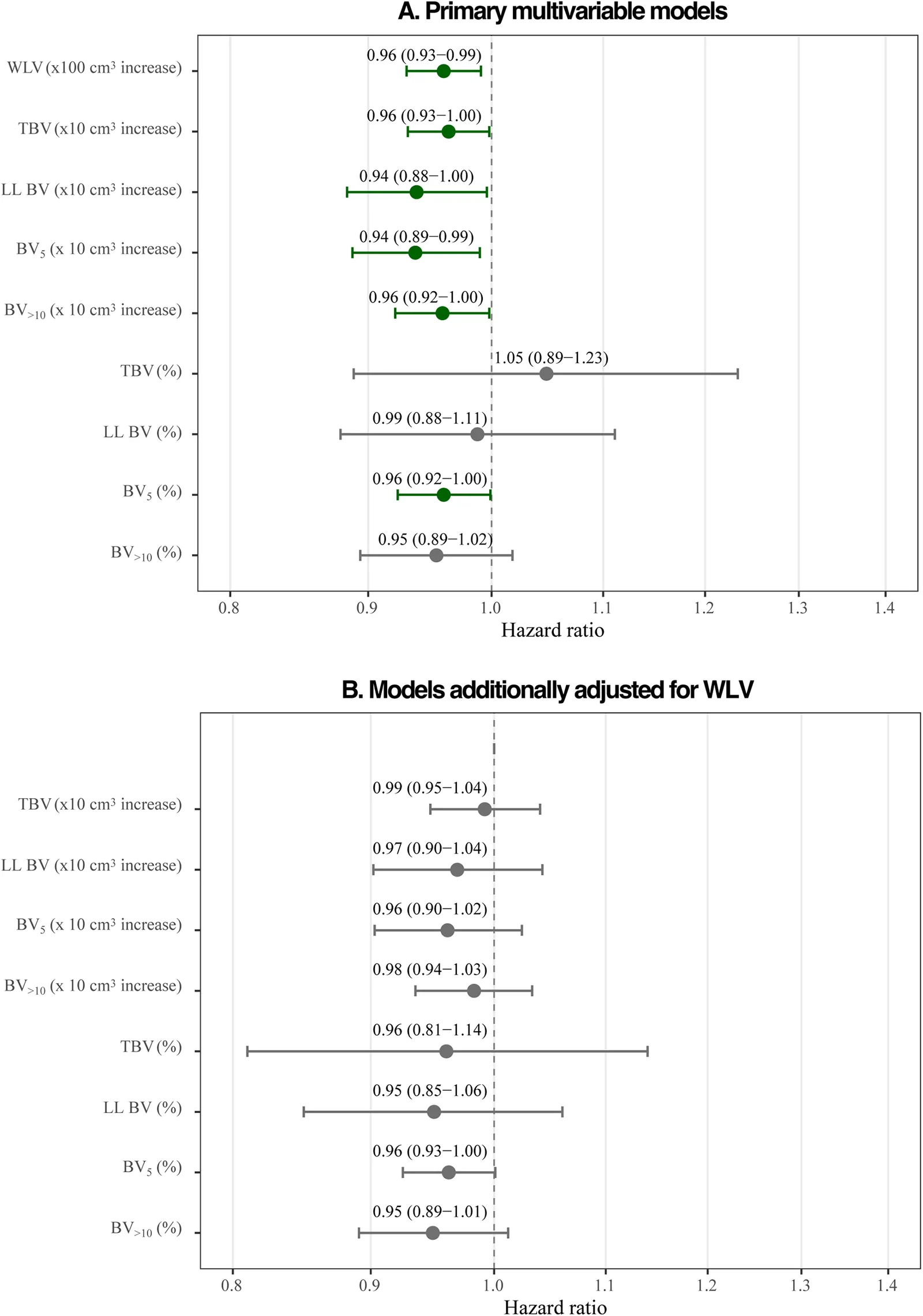
**(A)** Results of multivariable Cox regression models evaluating the associations of whole lung volume and pulmonary vascular metrics with mortality. Each row represents a separate multivariable Cox regression model including a single metric as the variable of interest and adjusted for cohort, age at diagnosis, sex, body mass index, GAP stage, usual interstitial pneumonia pattern, and fibrosis extent. **(B)** Pulmonary vascular metrics were evaluated in analogous models additionally including whole lung volume (WLV) as an adjustment covariate. Hazard ratios (HRs) and 95% confidence intervals (CIs) are shown for the metric of interest only. Hazard ratios for the adjustment covariates included in each model are reported in Supplementary Tables S7A,B, respectively. WLV, whole lung volume; TBV, total blood volume; BV, blood volume; LL, lower lobe.

## Discussion

In this dual-center retrospective cohort of newly diagnosed IPF, we found that lung volume-normalized and absolute pulmonary vascular metrics capture complementary, rather than interchangeable, aspects of disease. Lung volume-normalized vascular measures were strongly associated with fibrosis extent and baseline functional impairment, whereas absolute pulmonary vascular volumes, including TBV, BV_5_, and BV_>10_, were associated with survival in multivariable models, although these associations were substantially attenuated after accounting for WLV. Also, neither normalized nor absolute vascular metrics independently predicted longitudinal decline in FVC or DLCO. To derive these imaging biomarkers, we applied a fully automated deep learning-based quantitative CT approach integrating fibrosis burden and pulmonary vascular metrics. This approach enabled reproducible segmentation of pulmonary parenchyma and vascular structures despite the architectural distortion characteristic of fibrotic lung disease.

Vascular metrics have recently gained attention as a quantitative imaging biomarker across fibrotic ILD, with associations reported for fibrosis extent (9, 23), lung function decline (7, 9, 24, 25), disease progression, particularly in CTD-ILD, and mortality (6, 8, 15, 26–32). In line with prior quantitative CT findings (11), TBV was higher in IPF than in controls, with a mean normalized estimate of 8.3% corresponding to an average absolute TBV of 322 cm^3^. BV_5_ and BV_>10_ metrics were likewise increased, again as absolute and normalized measures. Reported TBV values in IPF vary widely across studies due to differences in populations and analytical approaches, ranging from 3.6 to 7.8% and absolute values from 60.9 to 270 cm^3^ (6–11, 15). Normalized TBV facilitates inter-individual comparisons but is sensitive to lung volume loss (“denominator effect”), whereas absolute TBV is less affected but varies with body size and baseline lung volume. In our data, only normalized TBV closely tracked fibrosis, reflecting the combined effect of fibrotic burden and volume loss, and therefore appearing to reflect structural severity rather than a disease-specific vascular signature. Supporting this, Gökçek et al. reported higher relative TBV in IPF and fibrotic hypersensitivity pneumonitis (fHP) than in nonspecific interstitial pneumonia, proposing discriminatory cut-offs (10). Consistently, vessel-related structure volume differed by diagnosis, and was higher in IPF than in CTD-ILD and fHP (8). Moreover, a relative TBV >6.5% in fHP has been shown to identify a subgroup with an IPF-like prognosis (33). However, these findings should be interpreted cautiously across ILD subtypes, as the biological meaning of CT-derived vascular metrics may differ between IPF and other fibrotic lung diseases. In CTD-ILD, vascular abnormalities may reflect both fibrosis-associated remodeling and disease-specific vascular injury, potentially influencing their prognostic implications. Beyond diagnostic discrimination, Chung et al. demonstrated that vessel-derived volume was the only quantitative metric independently associated with pathological UIP, with a 173 cm^3^ threshold performing comparably to qualitative CT assessment (34).

Consistent with prior reports (7, 9, 23), only lung-normalized vascular metrics correlated with baseline lung function, particularly DLCO, whereas absolute TBV did not. Baseline vascular metrics were not independently associated with subsequent functional decline. Previous longitudinal studies suggest that changes in vascular volumes over time, rather than baseline values, may better capture disease progression. Jacob et al. reported that longitudinal increases in TBV (absolute and relative), particularly in mid-lung regions, were associated with disease progression and FVC decline (27). Similarly, longitudinal quantitative analyses show that a ≥20% relative increase in vessel-related structure volume combined with declining FVC improves outcome stratification vs. visual scoring (35). Overall, baseline vascular metrics reflect structural state, whereas longitudinal changes better capture clinically meaningful progression, warranting prospective validation.

Previous studies have established associations between pulmonary vascular metrics and clinical outcomes in IPF but have generally evaluated these measures without directly addressing whether absolute and normalized approaches provide distinct biological information. Our study extends previous findings by demonstrating that the clinical associations of pulmonary vascular metrics depend on whether they are expressed as absolute or lung-volume normalized measures. Although both normalized and absolute TBV have been reported as independent mortality predictors in large IPF cohorts (6, 15), our multivariable models showed that absolute vascular metrics, including lower-lobe BV, BV_5_ and BV_>10_, together with WLV, were associated with improved survival, whereas normalized TBV was not. Importantly, the prognostic associations of absolute vascular metrics were substantially attenuated after additional adjustment for WLV, suggesting that their information is at least partly shared with lung volume. This finding does not necessarily conflict with prior CALIPER-based studies but may reflect the different biological information captured by relative and absolute vascular measures. Relative TBV appears to mainly reflect fibrosis extent, lung volume loss, and baseline functional impairment, whereas absolute TBV may reflect preserved lung volume and pulmonary reserve rather than an independent vascular prognostic effect. Therefore, the inverse association between relative TBV and lung function and the association between higher absolute TBV and improved survival should not be considered contradictory, as these metrics likely reflect different disease dimensions. The substantial overlap between absolute vascular volume and WLV in their associations with survival further supports this interpretation. The stronger association observed in the lower lobes and peripheral regions, which are typically affected by fibrosis, may indicate that regional preservation of vascular volume contributes to the observed prognostic association. In addition, BV_5_ and BV_>10_, as proxies of the distal and larger vessel compartments, respectively, may capture different aspects of vascular redistribution beyond global fibrosis assessment. Histopathology supports complex vascular remodeling in IPF not fully explained by pulmonary pressures, including medial thickening, luminal narrowing, and intimal hyperplasia within fibrotic regions (36). These changes may reflect chronic hypoxia and altered vascular signaling, suggesting that vasculopathy may contribute to fibrosis rather than simply represent a secondary phenomenon (37). Overall, associations between CT-derived pulmonary vascular metrics and hemodynamic surrogates in IPF and CTD-ILD remain inconsistent, precluding firm conclusions (7, 9, 11, 18, 23, 29, 38, 39). Therefore, the association between absolute vascular volumes and survival should be considered hypothesis-generating and requires external validation in independent IPF cohorts using harmonized CT acquisition, segmentation, and vascular quantification methods.

Several limitations should be noted. The retrospective design may introduce selection bias, and cohort differences in clinical characteristics could leave residual heterogeneity despite adjustment. Moreover, because height was not available, we were unable to directly account for one of the major determinants of thoracic size and pulmonary vascular volume. Consequently, some degree of residual confounding related to body habitus may persist, particularly for analyses involving absolute vascular measures. Generalizability is limited by restrictive eligibility criteria and exclusion of suboptimal CT scans. The relatively strict inclusion criteria, largely driven by predefined CT quality requirements and completeness of clinical data, resulted in a substantial exclusion rate; although this may introduce a degree of selection bias, it was necessary to ensure the robustness and reliability of quantitative CT-derived measurements in a deep learning-based imaging study. Although acquisition and reconstructions protocols were standardized, imaging data were obtained using five CT platforms from multiple vendors, and no formal quantitative assessment of scanner effects or *post-hoc* harmonization procedure was performed. This was mainly due to the retrospective nature of the study and the limited number of patients per scanner platform, which would have made scanner-specific modeling underpowered and potentially unstable. To reduce technical variability, all examinations were processed using the same fully automated vendor-integrated pipeline, thereby ensuring methodological consistency across datasets. Furthermore, the evaluated vascular metrics were derived from anatomically constrained vessel segmentation and morphometric quantification rather than conventional intensity-based radiomic features, which are generally more sensitive to acquisition-related variability. Nevertheless, residual scanner-dependent effects cannot be completely excluded. In addition, reliance on a single proprietary platform with expert correction may limit transferability, particularly to studies using alternative software frameworks such as CALIPER. Longitudinal lung-function analyses included only cases with follow-up, introducing potential attrition bias. Importantly, the present study was designed to evaluate independent associations with mortality rather than the incremental prognostic value of pulmonary vascular metrics beyond established clinical predictors such as GAP score, FVC, DLCO, or CT-derived fibrosis extent.

Although multivariable Cox models were used to evaluate independent associations with mortality, formal assessment of added predictive value would require a dedicated model-comparison framework. Therefore, the clinical utility of these metrics for improving risk stratification remains to be established. Finally, the absence of systematic invasive hemodynamics precludes mechanistic conclusions regarding CT-derived vascular metrics and pulmonary hypertension.

In conclusion, deep learning-based quantitative CT shows that absolute and lung-volume normalized pulmonary vascular metrics are not interchangeable imaging biomarkers in IPF. Lung volume-normalized measures were more closely associated with fibrosis burden and functional impairment, whereas absolute vascular volumes were associated with mortality in multivariable models, although these associations were substantially attenuated after accounting for WLV. This attenuation suggests that the prognostic information provided by absolute vascular volume may partly reflect preserved lung volume rather than an independent vascular effect. The clinical significance and incremental prognostic value of these imaging biomarkers beyond established clinical models warrant prospective evaluation.

## Data Availability

The original contributions presented in the study are included in the article/Supplementary Material, further inquiries can be directed to the corresponding author/s.
