# Supplementary material for: Absolute and lung volume-normalized deep learning-derived pulmonary vascular measures in idiopathic pulmonary fibrosis: distinct clinical associations and the role of lung volume in prognosis
Source: Front Med (Lausanne). 2026 Sep 29;13:1833470. doi: 10.3389/fmed.2026.1833470 (PMC13658197; doi:10.3389/fmed.2026.1833470)
Supplement: Supplementary file 1 [file Datasheet1.pdf]

Supplementary Table 1. CT acquisition and reconstruction protocols across the five scanner platforms used in the study

Parameter	Siemens Somatom Force	Siemens Somatom Drive	GE LightSpeed VCT (64-slice)	Toshiba Aquilion One	Philips Brilliance (64-slice)
**Detector configuration**	2 × 96 × 0.6 mm	2 × 64 × 0.6 mm	64 × 0.625 mm	320 × 0.5 mm	64 × 0.625 mm
**Tube voltage (kVp)**	120	120	120	120	120
**Tube current (mAs)**	Auto mA (CARE Dose4D)	Auto mA (CARE Dose4D)	Auto mA (SmartmA)	Auto mA (SureExposure 3D)	Auto mA (DoseRight)
**Rotation time (s)**	0.25	0.28	0.4	0.35	0.4
**Pitch factor**	0.90-1.20	0.90-1.15	0.984	Volumetric (Axi-Scanned) / 0.813	0.924
**Acquisition direction**	Craniocaudal	Craniocaudal	Craniocaudal	Craniocaudal	Craniocaudal
**Inspiratory phase**	Full inspiration	Full inspiration	Full inspiration	Full inspiration	Full inspiration
**Slice thickness (mm)**	0.6	0.6	0.625	0.5	0.625
**Reconstruction interval (mm)**	0.5	0.5	0.625	0.5	0.5
**Reconstruction matrix**	1024 × 1024	1024 × 1024	512 × 512	512 × 512	512 × 512
**Reconstruction Kernel (Lung)**	Bl57 / Bl64 (Ultra-sharp)	Bl57 / Bl64 (Ultra-sharp)	Lung / Bone	FC52 / FC56 (Sharp)	Y-Sharp (Y-B)
**Reconstruction Kernel (Soft)**	Br40 (Medium Smooth)	Br40 (Medium Smooth)	Standard	FC03 / FC08 (Standard)	Standard (B)
**Iterative reconstruction**	ADMIRE (Level 3)	ADMIRE (Level 3)	ASiR (40%)	AIDR 3D	iDose4 (Level 3)

Acronyms: kVp, kilovoltage peak; mAs, milliampere-seconds; mm, millimeters; s, seconds; HRCT, high-resolution computed tomography.

Supplementary Table 2. Quantitative assessment of fibrosis extent and vascular metrics per lung lobe

A)

Fibrosis extent	Overall population n=118 (100%)	Italian cohort n=57 (48.3%)	French cohort n=61 (51.7%)
**RUL, %**	8 [3; 15] (0 to 55)	8 [4; 18.8] (0 to 55)	8 [2; 11.5] (0 to 31)
**LUL, %**	6 [3; 14] (0 to 44)	8 [4; 23] (0 to 44)	4 [2; 11] (0 to 25)
**ML, %**	8 [3; 17] (0 to 67)	8.5 [3.2; 18] (0 to 67)	7 [2; 14.5] (0 to 56)
**RLL, %**	19 [9; 28] (1 to 80)	21 [13; 39.8] (4 to 79)	15 [6.5; 24] (1 to 80)
**LLL, %**	18 [8; 32] (0 to 88)	27.5 [11.2; 39] (1 to 88)	14 [5; 24.5] (0 to 79)

B)

Vascular metrics	Overall population n=118 (100%)	Italian cohort n=57 (48.3%)	French cohort n=61 (51.7%)
**RUL BV, cm^3**	69.9±22.3 (13 to 131)	67.6±24.4 (13 to 131)	71.9±20.3 (26 to 126)
**LUL BV, cm^3**	70±25.6 (11 to 134)	62.6±26 (11 to 129)	76.6±23.5 (12 to 134)
**ML BV, cm^3**	18.3±12.9 (0 to 49)	14.5±12.7 (0 to 39)	21.7±12.3 (2 to 49)
**RLL BV, cm^3**	87±31.3 (14 to 185)	83.6±32.7 (14 to 185)	90±29.9 (28 to 162)
**LLL BV, cm^3**	77.3±25.1 (18 to 155)	74.4±25.2 (18 to 132)	80±24.9 (24 to 155)
**LL BV, cm^3**	164.3±54.2 (32 to 317)	158±55.4 (32 to 317)	170±52.9 (52 to 306)
**UL BV, cm^3**	139.8±45.1 (27 to 248)	130.2±47 (27 to 233)	148.5±41.7 (38 to 248)
**LL/UL BV (cm^3) ratio**	1.2±0.3 (0.6 to 3.5)	1.3±0.4 (0.6 to 3.5)	1.2±0.2 (0.6 to 1.8)
**RUL BV, %**	10.5±4.1 (3.2 to 25.9)	10.6±4.8 (3.2 to 25.9)	10.5±3.2 (3.6 to 18.6)
**LUL BV, %**	7.5±2.2 (2.1 to 12.8)	7.4±2.5 (2.1 to 12.8)	7.6±1.9 (3.1 to 11.8)
**ML BV, %**	4.5±2.9 (0 to 12.2)	4.2±3.2 (0 to 12.1)	4.8±2.5 (0.5 to 12.2)
**RLL BV, %**	8.7±3.9 (2.1 to 25.1)	9±4.2 (2.1 to 25.1)	8.5±3.6 (3.4 to 19.3)
**LLL BV, %**	8.7±3.4 (2.5 to 22.5)	9.2±3.9 (2.5 to 22.5)	8.3±2.9 (3.9 to 19.4)
**LL BV, %**	8.6±3.2 (2.3 to 18.4)	8.9±3.5 (2.3 to 18.4)	8.3±3 (3.7 to 18)
**UL BV, %**	8.7±2.7 (2.5 to 15.1)	8.7±3.1 (2.5 to 15.1)	8.7±2.3 (3.5 to 14.2)
**LL/UL BV (%) ratio**	1±0.3 (0.5 to 2.6)	1.1±0.3 (0.5 to 2.6)	1±0.2 (0.6 to 1.4)

Data are presented as mean ± standard deviation (SD) and range (min-max), or as median [interquartile range] and range (min-max), where appropriate.

Acronyms: RUL, right upper lobe; LUL, left upper lobe; ML, middle lobe; RLL, right lower lobe; LLL, left lower lobe; BV, blood volume; LL, lower-lobe; UL, upper-lobe.

Supplementary Table 3. Correlations of blood volume and fibrosis extent per lung lobe

**Fibrosis score, %**	**BV, % (*p* value)**	**BV, cm^3 (*p* value)**
**RUL**	0.506 (**<0.001**)	0.131 (0.16)
**LUL**	0.242 (**0.009**)	-0.127 (0.175)
**ML**	-0.053 (0.58)	-0.148 (0.112)
**RLL**	0.702 (**<0.001**)	0.35 (**<0.001**)
**LLL**	0.655 (**<0.001**)	0.158 (0.091)

Data are presented as Pearson's correlation coefficient. Associations with a *p* value < 0.05 were considered statistically significant and reported in bold.

Acronyms: BV, blood volume; RUL, right upper lobe; LUL, left upper lobe; ML, middle lobe; RLL, right lower lobe; LLL, left lower lobe.

Supplementary Table 4. Pairwise correlations among whole lung volume and pulmonary vascular metrics.

	WLV (cm^3)	TBV (cm^3)	LL BV (cm^3)	BV_5 (cm^3)	$BV_{>10}$ (cm^3)	TBV (%)	LL BV (%)	BV_5 (%)	$BV_{>10}$ (%)
WLV (cm^3)	1	**0.505 (<0.001)**	**0.365 (<0.001)**	**0.344 (<0.001)**	**0.435 (<0.001)**	**-0.417 (<0.001)**	**-0.423 (<0.001)**	-0.016 (0.864)	-0.123 (0.187)
TBV (cm^3)		1	**0.916 (<0.001)**	**0.928 (<0.001)**	**0.983 (<0.001)**	**0.53 (<0.001)**	**0.457 (<0.001)**	**0.486 (<0.001)**	**0.331 (<0.001)**
LL BV (cm^3)			1	**0.865 (<0.001)**	**0.91 (<0.001)**	**0.6 (<0.001)**	**0.626 (<0.001)**	**0.465 (<0.001)**	**0.335 (<0.001)**
BV_5 (cm3)				1	**0.978 (<0.001)**	**0.605 (<0.001)**	**0.543 (<0.001)**	**0.754 (<0.001)**	**0.623 (<0.001)**
$BV_{>10}$ (cm^3)					1	**0.577 (<0.001)**	**0.507 (<0.001)**	**0.619 (<0.001)**	**0.485 (<0.001)**
TBV (%)						1	**0.944 (<0.001)**	**0.525 (<0.001)**	**0.447 (<0.001)**
LL BV (%)							1	**0.489 (<0.001)**	**0.428 (<0.001)**
BV_5 (%)								1	**0.943 (<0.001)**
$BV_{>10}$ (%)									1

Data are presented as Pearson's correlation coefficient. Associations with a p value < 0.05 were considered statistically significant and reported in bold.

Acronyms: WLV, whole lung volume; TBV, total blood volume; BV, blood volume; LL, lower lobes.

Supplementary Table 5. Correlations of vascular metrics with lung function at baseline

Parameter	FVC, % pred	$DLCO_{sb}$, % pred
**WLV, cm^3**	0.323 (**<0.001**)	0.471 (**<0.001**)
**TBV, %**	-0.444 (**<0.001**)	-0.511 (**<0.001**)
**TBV, cm^3**	-0.105 (0.259)	-0.063 (0.496)
**BV_5, %**	-0.197 (**0.033**)	-0.242 (0**.008**)
**BV_5, cm^3**	-0.156 (0.094)	-0.151 (0.104)
**$BV_{>10}$, %**	-0.203 (**0.027**)	-0.241 (**0.009**)
**$BV_{>10}$, cm^3**	-0.129 (0.165)	-0.106 (0.257)

Data are presented as Pearson's correlation coefficient. Associations with a *p* value < 0.05 were considered statistically significant and reported in bold.

Acronyms: WLV, whole lung volume; TBV, total blood volume; BV, blood volume; FVC, forced vital capacity; $DLCO_{sb}$, diffusion capacity of the lung for carbon $monoxide_{single\text{-}breath.}$

Supplementary Table 6. Univariable Cox proportional hazards regression analysis of predictors of mortality

Variable	HR (95% CI)	*p* value
**Cohort, Italian**	1.39 (0.87 to 2.24)	0.169
**Sex, male**	1.24 (0.7 to 2.17)	0.459
**Age at diagnosis, years**	1.02 (0.99 to 1.05)	0.241
**Smoking, yes**	0.91 (0.53 to 1.56)	0.738
**BMI, kg/m^2**	1.02 (0.97 to 1.08)	0.445
**Dyslipidemia**	0.48 (0.29 to 0.81)	**0.006**
**Ischemic heart disease**	0.61 (0.34 to 1.13)	0.115
**Systemic arterial hypertension**	0.92 (0.58 to 1.47)	0.731
**Type II diabetes**	0.61 (0.36 to 1.05)	0.073
**Therapy**		
**None (ref)**	-	
**Nintenadib**	1.79 (0.71 to 4.49)	0.217
**Pirfenidone**	2.24 (0.93 to 5.41)	0.073
**Both**	1.85 (0.72 to 4.72)	0.201
**GAP stage (ref I)**		
**II**	3.46 (2.06 to 5.81)	**<0.001**
**III**	4.46 (2.12 to 9.39)	**<0.001**
**Chest CT pattern; probable UIP (ref)**	0.36 (0.22 to 0.57)	**<0.001**
**WLV, cm^3**	0.95 (0.93 to 0.98)	**<0.001**
**Total fibrosis extent, %**	1.06 (1.04 to 1.07)	**<0.001**
**URL, %**	1.04 (1.02 to 1.06)	**<0.001**
**LUL, %**	1.05 (1.03 to 1.07)	**<0.001**
**ML, %**	1.03 (1.02 to 1.05)	**<0.001**
**RLL, %**	1.03 (1.02 to 1.04)	**<0.001**
**LLL, %**	1.03 (1.02 to 1.05)	**<0.001**
**TBV, cm^3**	1.02 (0.79 to 1.31)	0.889
**URL, cm^3**	1.15 (0.37 to 3.57)	0.81
**LUL, cm^3**	0.7 (0.26 to 1.9)	0.484
**ML, cm^3**	0.25 (0.03 to 1.73)	0.159
**RLL, cm^3**	1.4 (0.68 to 2.89)	0.359
**LLL, cm^3**	1.28 (0.5 to 3.31)	0.605
**LL, cm^3**	1.02 (0.97 to 1.06)	0.438
**UL, cm^3**	0.99 (0.94 to 1.05)	0.781
**LL/UL BV (cm^3) ratio**	2.58 (1.26 to 5.3)	**0.010**
**TBV, %**	1.22 (1.11 to 1.33)	**<0.001**
**URL, %**	1.07 (1.01 to 1.13)	**0.018**
**LUL, %**	1.18 (1.05 to 1.32)	**0.006**
**ML, %**	1.03 (0.94 to 1.13)	0.524
**RLL, %**	1.09 (1.04 to 1.14)	**<0.001**

**LLL, %**	1.15 (1.08 to 1.22)	**<0.001**
**LL, %**	4.01 (2.11 to 7.6)	**<0.001**
**UL, %**	3.69 (1.45 to 9.37)	**0.006**
**LL/UL BV (%) ratio**	4.18 (1.75 to 10.02)	**0.001**
**BV_5, cm^3**	1.01 (0.97 to 1.05)	0.582
**$BV_{>10}$, cm^3**	1.01 (0.98 to 1.04)	0.601
**BV_5, %**	1.01 (0.98 to 1.04)	0.594
**$BV_{>10}$, %**	1.02 (0.97 to 1.07)	0.45

Variables with a *p* value < 0.05 were considered statistically significant and reported in bold.

Acronyms: HR, hazard ratio; CI, confidence interval; BMI, body mass index; GAP, Gender-Age-Physiology; UIP, usual interstitial pneumonia; WLV, whole lung volume; TBV, total blood volume; RUL, right upper lobe; LUL, left upper lobe; ML, middle lobe; LL, lower-lobe; UL, upper-lobe; BV, blood volume.

Supplementary Table 7. Hazard ratios of adjustment covariates across multivariable Cox models evaluating pulmonary vascular metrics.

Predictor	WLV (cm^3)	TBV (cm^3)	LL BV (cm^3)	BV_5 (cm^3)	$BV_{>10}$ (cm^3)	TBV (%)	LL BV (%)	BV_5 (%)	$BV_{>10}$ (%)
**Cohort, Italian**	0.83 (0.48 to 1.45)	0.71 (0.4 to 1.27)	0.67 (0.37 to 1.21)	0.58 (0.3 to 1.1)	0.65 (0.36 to 1.19)	0.87 (0.48 to 1.58)	0.79 (0.43 to 1.44)	0.54 (0.26 to 1.12)	0.62 (0.3 to 1.26)
**Gender, male**	1.4 (0.69 to 2.85)	1.3 (0.65 to 2.62)	1.1 (0.56 to 2.14)	1.23 (0.62 to 2.44)	1.23 (0.62 to 2.46)	0.93 (0.49 to 1.77)	0.91 (0.48 to 1.75)	0.97 (0.51 to 1.87)	0.91 (0.47 to 1.74)
**Age at diagnosis, years**	1.01 (0.98 to 1.04)	1 (0.97 to 1.03)	1 (0.97 to 1.03)	1 (0.97 to 1.03)	1 (0.97 to 1.03)	1.01 (0.97 to 1.04)	1.01 (0.97 to 1.04)	1 (0.97 to 1.04)	1 (0.97 to 1.04)
**Fibrosis extent, %**	1.02 (0.99 to 1.05)	**1.05 (1.02 to 1.08)**	**1.05 (1.01 to 1.08)**	**1.05 (1.02 to 1.08)**	**1.05 (1.01 to 1.08)**	1.02 (0.98 to 1.06)	1.03 (0.99 to 1.07)	**1.05 (1.01 to 1.08)**	**1.04 (1.01 to 1.07)**
**Chest CT pattern, probable UIP (ref)**	**0.51 (0.27 to 0.96)**	0.58 (0.31 to 1.06)	**0.53 (0.28 to 0.99)**	**0.54 (0.29 to 1)**	0.54 (0.29 to 1.01)	0.6 (0.32 to 1.13)	0.62 (0.33 to 1.15)	0.59 (0.32 to 1.09)	0.61 (0.33 to 1.12)
**BMI, kg/m^2**	1.03 (0.97 to 1.09)	1.01 (0.96 to 1.07)	1.02 (0.96 to 1.08)	1.01 (0.95 to 1.07)	1.01 (0.96 to 1.08)	1.02 (0.97 to 1.09)	1.03 (0.97 to 1.09)	1.01 (0.96 to 1.07)	1.02 (0.96 to 1.08)
**GAP score (ref I)**									
**II**	**2.03 (1.08 to 3.81)**	**2.02 (1.09 to 3.71)**	**2.23 (1.19 to 4.18)**	**2.15 (1.16 to 3.98)**	**2.15 (1.16 to 3.99)**	**2 (1.07 to 3.74)**	**2.02 (1.08 to 3.79)**	**2.07 (1.12 to 3.83)**	**2.05 (1.11 to 3.79)**
**III**	1.28 (0.47 to 3.54)	1.29 (0.47 to 3.51)	1.54 (0.57 to 4.17)	1.4 (0.52 to 3.8)	1.43 (0.52 to 3.9)	1.97 (0.76 to 5.06)	2.16 (0.83 to 5.58)	1.98 (0.78 to 5.05)	2.07 (0.81 to 5.26)

Column headings identify the pulmonary vascular metric included as the predictor of interest in each model. Statistically significant associations ($p < 0.05$) are shown in bold. Hazard ratios for the vascular metrics themselves are presented in Figure 3.

Acronyms: UIP, usual interstitial pneumonia; BMI, body mass index; GAP, Gender-Age-Physiology; WLV, whole lung volume; TBV, total blood volume; LL, lower-lobe; BV, blood volume.
